# A high-resolution streamflow and hydrological metrics dataset for ecological modeling using a regression model

**DOI:** 10.1038/sdata.2018.224

**Published:** 2018-11-06

**Authors:** Katie Irving, Mathias Kuemmerlen, Jens Kiesel, Karan Kakouei, Sami Domisch, Sonja C. Jähnig

**Affiliations:** 1 Department of Ecosystem Research, Leibniz-Institute of Freshwater Ecology and Inland Fisheries (IGB), Müggelseedamm 310, 12587 Berlin, Germany; 2 Department of Biology, Chemistry and Pharmacy, Freie University Berlin, Takustraße 3, 14195 Berlin, Germany; 3 Department Systems Analysis, Integrated Assessment and Modeling, Eawag, Überlandstrasse 133, CH-8600 Dübendorf, Switzerland; 4 Christian-Albrechts-University Kiel, Institute for Natural Resource Conservation, Department of Hydrology and Water Resources Management, Kiel, Germany

**Keywords:** Freshwater ecology, Ecological modelling, Hydrology

## Abstract

Hydrological variables are among the most influential when analyzing or modeling stream ecosystems. However, available hydrological data are often limited in their spatiotemporal scale and resolution for use in ecological applications such as predictive modeling of species distributions. To overcome this limitation, a regression model was applied to a 1 km gridded stream network of Germany to obtain estimated daily stream flow data (m^3^ s^−1^) spanning 64 years (1950–2013). The data are used as input to calculate hydrological indices characterizing stream flow regimes. Both temporal and spatial validations were performed. In addition, GLMs using both the calculated and observed hydrological indices were compared, suggesting that the predicted flow data are adequate for use in predictive ecological models. Accordingly, we provide estimated stream flow as well as a set of 53 hydrological metrics at 1 km grid for the stream network of Germany. In addition, we provide an R script where the presented methodology is implemented, that uses globally available data and can be directly applied to any other geographical region.

## Background & Summary

Natural flow regimes have a large influence in shaping biological communities^1^ and regulate numerous ecological processes in stream ecosystems^2–4^. Future flow regimes are predicted to alter significantly by, for example, an increase in the frequency and severity of floods and droughts^5,6^. To understand the ecological consequences of these changes, it is important to study how the critical components of flow regimes affect stream ecosystems and to include this knowledge in the assessment of future global-change scenarios. However, information about flow regimes is often not available or sufficiently diverse for detailed modeling analyses such as species distribution models (SDMs)^7,8^, which are a common tool used in ecological analysis. SDMs relate known occurrences of species to their environmental conditions and predict species distributions in geographical space under current or future conditions.

One way to include flow regime information into SDMs, is the implementation of the “Indicators of Hydrologic Alteration” (IHA)^9^, which describe the magnitude, frequency, duration, timing and change rate of high, low and average stream flow conditions. The metrics can provide essential information on freshwater ecosystems in general and on the impact of human activities and may support river management and conservation. Additionally, they are well suited to be applied in predictive modeling (i.e. SDMs) and can be used under future hydrological scenarios to assess the effects of climate change on species distribution.

While the tools required to calculate IHA are freely available (i.e. www.github.com/USGS-R/EflowStats)^10,11^, the stream flow data required for carrying out ecological predictions can be challenging to acquire, as they needs to be of high temporal resolution (daily, m^3^ s^−1^), continuous (i.e. gapless in time and space) and regionally representative. Such data are typically restricted to gauging stations i.e. only point localities being available for analysis, therefore it is not possible to analyze large sections of a stream network. An effective way to obtain spatially gapless data is through the application of hydrological models. However, predictive modeling applications such as SDMs in rivers often require environmental predictors at fine spatial resolutions (<1 km^2^), over a large spatial scale, in order to include the species’ full range of occurrence for comprehensive predictions^12^. Depending on the complexity of hydrological model (such as SWAT^13^, WaSIiM-ETH^14^) and the large amount of input data required, it can become tedious to simulate on these spatial scales. Given these limitations, in order to fill the much-needed data gap for ecological analyses, linear regression models are simple and fast methods that can be applied for the spatiotemporal extrapolation of stream flow^15,16^. Following these considerations, the regression model developed here used two freely available data components: 1) observed gauging data from the Global Runoff Data Centre (GRDC, www.bafg.de/GRDC/), 2) downstream accumulated precipitation data along a river network at high resolution^17^. The low data requirements of our model, together with the simple modeling approach, render it an inexpensive, easy-to-use tool which can be applied to any geographical scale, and/or time period.

We applied the model to a 1 km gridded stream network in Germany (n=85,363 1 km grid cells) to create a continuous daily time series of stream flow (m^3^ s^−1^) spanning 64 years. The estimated daily stream flow data were used as input to create a set of IHA metrics for the German stream network. From the 165 metrics that were tested, 53 were validated successfully: predominantly metrics describing mean values of stream flow, e.g. mean monthly flow.

To test the results for their usability in ecological applications, we predicted the occurrence of 34 benthic macroinvertebrate species with GLM models using the validated metrics with either observed or simulated values. Results from both yielded equally good predictions, showing that data predicted from this study is adequate for the purpose of ecological predictive modeling.

We provide both the simulated daily stream flow dataset and the 53 IHA metrics as downloadable files (Data Citation 1) which can be used “as is”. In addition, we provide R scripts that allow users to apply the model to other geographical regions or calculate the hydrological metrics for different time periods.

## Methods

The primary goal of this study was to create a much-needed dataset of hydrological variables for use in ecological predictive modeling. There is a high demand^18^ for large scale hydrological data at high spatiotemporal resolutions to be used as input in models such as species distribution models. Such data should be either widely available or easy to reproduce. For this reason, we propose to estimate stream flow for entire stream networks using a linear regression model with only one predictor: the accumulative precipitation in the upper subcatchment. While there are several other relevant processes influencing discharge (e.g. infiltration, groundwater storage, evapotranspiration, etc.), adding further predictors significantly increases model complexity and computation time, particularly if models are of large scale and high spatiotemporal resolution. We are aware that applying a simple model over a large scale and fine resolution comes at the cost of lower prediction accuracy, as it does not take into account important natural and anthropogenic influences (e.g. water abstraction and river management such as dams). Nevertheless, a model which can be readily implemented will help fill the demand for large scale, continuous, high spatiotemporal resolution data to be used in ecological predictive modeling, specifically SDMs.

**Figure 1:**
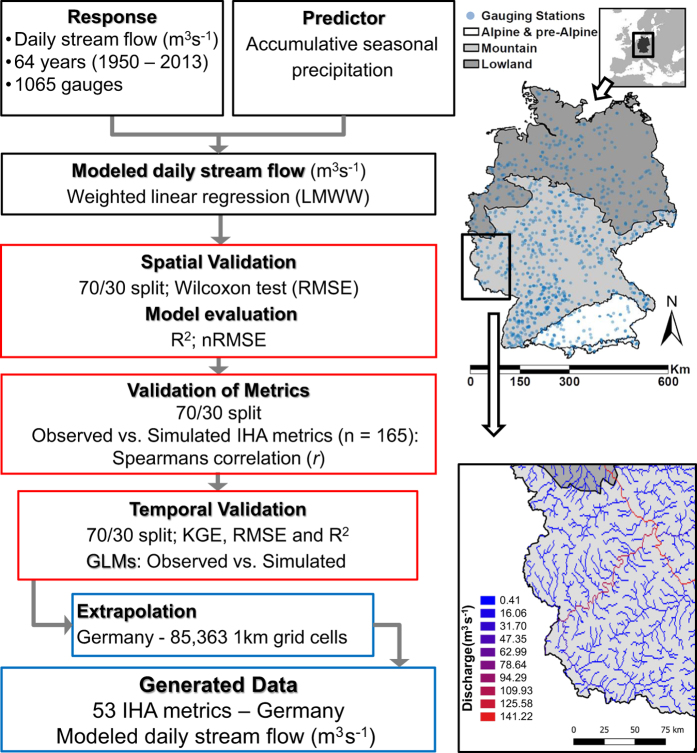
Workflow of the modeling and validation procedure. Observed daily stream flow data was used as the response variable in a weighted linear regression (LMWW). Seasonal accumulative precipitation data was used as the predictor variable. The model was validated spatially using Wilcoxon tests on the RMSE. The IHA metrics were calculated from observed and simulated streamflow and validated through Spearman’s correlation. The model was validated temporally with KGE, RMSE and R^2^, and GLMs were performed on 34 species and compared with IHA metrics calculated from observed and simulated flow data, respectively. The daily stream flow data were extrapolated to the entire stream network of Germany resulting in a time series covering 64 years. The validated IHA indices were then calculated for all grid cells on the stream network. Top right map is Germany’s location in Europe, and underneath, the study area of Germany with distribution of gauging stations. Bottom right map shows the modeled daily stream flow (m^3^s^−1^) of 11^th^ February 1950.

Moreover, our proposed modeling framework should be easily applied globally at any scale, which is why open and near global data sources have been chosen. Here, observed daily stream flow is extrapolated from gauging stations to all grid cells on the German stream network using weighted linear regression and subcatchment-accumulated precipitation as a predictor. The predicted daily stream flow data was used as input data to calculate the IHA metrics set out in Olden and Poff (2003)^19^ for every grid cell (see Fig. 1).

In a last step, the level of accuracy of the model predictions for the purpose of ecological modeling were tested by implementing Generalized Linear Models (GLMs) and comparing species occurrence predictions of 34 stream macroinvertebrates at gauging stations using both observed and predicted stream flow.

### Base layer & study area

The area of study is the stream network of Germany. An openly available, modeled 1 km gridded stream network of Germany was used as a base layer, taken from earthEnv.org/streams (2016)^17^, originally derived from the modeled HydroSHEDS dataset (www.hydrosheds.org, 30-arc-second spatial grain^20^), which in turn is derived from SRTM (http://srtm.csi.cgiar.org/)^21^ and available  in GEOTiff raster file format (Fig. 1).

### Data collection: stream flow

Daily stream flow data (m^3^ s^−1^) were collected from 1,065 gauging stations in Germany from the GRDC and the Federal Environment Agencies of Germany (Table 1). While all 1,065 sites collectively covered 64 years, each individual site had to contain at least 10 years of continuous data between 1^st^ January 1950 until 31^st^ December 2013 to be considered in order to maximize input and to standardize the dataset. Due to possible simplifications in the 1 km stream network, the gauging station sites within a 3 km buffer were moved to the next grid cell of the stream network base layer in QGIS (www.qgis.org/en/site/)^22^; any stations beyond the buffered network were excluded from the analysis.

### Data collection: predictors

We wanted to use freely and globally available data as model predictors, as we aim to produce a framework that can be applied in other geographical regions. The predictors had to also match the high spatial resolution of 1 km grid cells to be able to be applied.

Subcatchment-accumulated precipitation (hereafter referred to as precipitation) data were taken from www.earthenv.org/streams (2016)^17^, which in turn was derived from WorldClim database^23^. It consists of 12 average monthly downstream precipitation values (Jan–Dec) for each grid cell along the stream network, where each monthly value is a 50 year average (1950–2000)^17^. The data in raster format represent the stream network of the study area outlined above^24^. In this dataset, each grid cell of the stream network represents the added precipitation of all grid cells contained in the contributing area (i.e. upper subcatchment) of that specific cell. The accumulative nature of the data takes into account influencing upstream processes, important for stream systems^25^.

The predictor variable precipitation was determined by calculating the mean monthly precipitation of four sets of three months to represent the annual seasons: Dec–Feb (Winter), Mar-May (Spring), Jun-Aug (Summer) and Sep-Nov (Fall). The stream flow data began on 1^st^ Jan 1950 and ended on 31^st^ Dec 2013. Therefore, the seasonal precipitation for winter of 1950 consisted only of Jan and Feb, with Dec 1949 being omitted. Each winter thereafter included all 3 months (Dec–Feb) and is referred to as winter of the year containing the information from January and February (i.e. Dec 1983 + Jan and Feb 1984 = winter 1984). Similarly, only data from December 2013 were available for winter 2014.

### Preliminary analysis

The preliminary analysis tested model performance of various configurations of predictors and spatial scale, the purpose of which was to find the better performing model at the spatial scale and resolution we required. Therefore, we ran the analysis by applying three different predictors in the regression model: 1) flow accumulation, 2) monthly precipitation and 3) seasonal precipitation. The model trials were then applied on four different regional extents as to detect regional differences in model performance: 1) Germany in its entirety, 2) Germany sub divided into three regions i.e. Central Plains, Central Highlands and Alpine^26^. This procedure resulted in 12 model configurations in total, which were compared in their capability of predicting daily stream flow.

Flow accumulation is the sum of contributing grid cells from the upper subcatchment that naturally flow into one grid cell and is known to be highly correlated with stream flow^27,28^. Flow accumulation was taken from www.earthenv.org/streams, based on the HydroSHEDS digital elevation model (DEM)^20^, which in turn is derived from SRTM^21^. Here, flow direction was used as a base layer for routing and delineating the upper stream network in grass GIS (see Domisch *et al.* 2015^17^ for further details). It is important to note that due to the precipitation variables being accumulative, flow accumulation information is contained within the precipitation. This induces correlation between both and therefore the variables were tested in separate models.

Three performance metrics were used to compare model performance and validate the model: 1) Root mean square error (RMSE), 2) normalized root mean square error (nRMSE) and 3) coefficient of determination (R^2^). The R^2^ of each regression model was used for evaluation and to compare between model configurations (reported as mean R^2^ +/− SE) and referred to as explained variance. Both RMSE and nRMSE are measurements of each model’s predictive ability and were calculated from the observed and simulated flow values using the HydroGof package^29^. It is important to note that the performance metrics are derived from the regression model applied on each individual daily time step and were used to compare between each model configuration. This is a comparison of model goodness of fit, which was used to assess the model spatially across ecoregions and using different predictors. To effectively visualize the comparison between model configurations/spatial regions with differing variations of stream flow and orders of magnitude, the RMSE was normalized (nRMSE). This was calculated manually by dividing the RMSE by the difference in the maximum and minimum observed stream flow values.

Wilcoxon tests were applied to compare the RMSE values of each model configuration. Any individual dataset was large (n = 23,376 days), so in order to avoid possible type 1 errors (false positives), the data were split into yearly subsets (n = 365 ^∗^64). Each set of 64 Wilcoxon tests are reported as a percentage (%) of tests that show a significant difference between the two predictions tested. A higher percentage of significance indicates a larger difference between configurations. For comparison, each configuration was also tested using the entire dataset (n=23,376 days), without sub-dividing into annual components.

**Figure 2:**
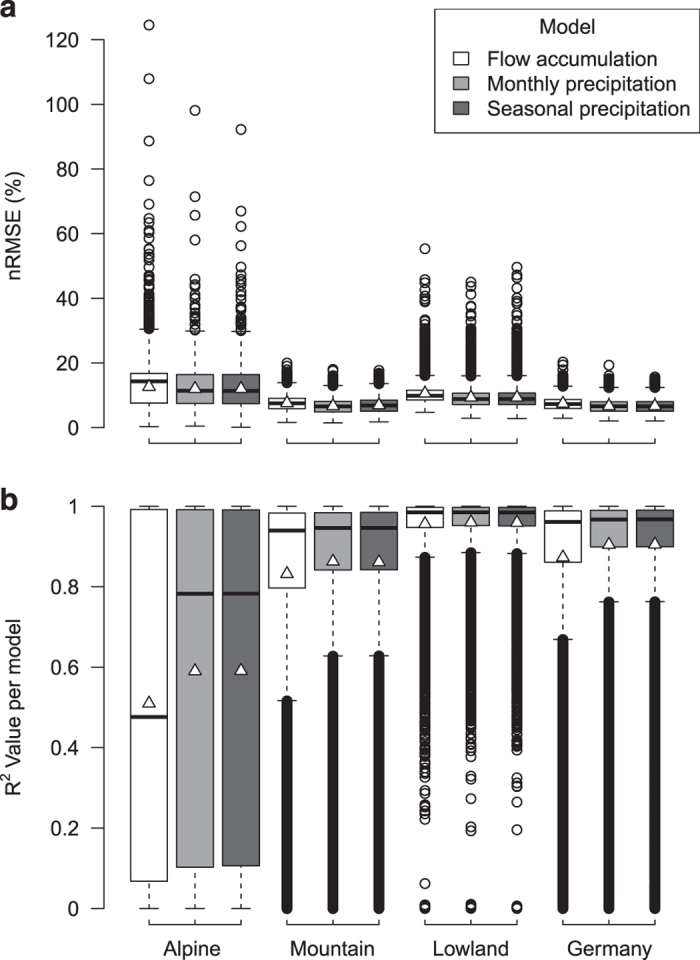
Comparison between each model configuration. Performance metric (**a**) normalized rooted mean square error (nRMSE) and (**b**) R squared statistic (R^2^). Points represent each model (day) over the time series (n = 23,376 models). Boxplots (bar = median, box = IQR, whiskers = 1.5×IQR and outliers. Triangles = mean (Table 2).

Precipitation performed better as a predictor than flow accumulation for both nRMSE (Fig. 2a,Table 2) and R^2^ (Fig. 2b, Table 2) across all regions. There was no significant difference between monthly and seasonal precipitation predictors (Fig. 2b, Wilcoxon Test: Table 3), with the only exception in the mountain region. Models applied with flow accumulation only performed significantly worse than any of the precipitation predictors (Wilcoxon Test: Table 3) and were not applied in further analyses. Models in the lowland region had the highest R^2^ values (mean R^2^=0.96, Table 2, Fig. 2a), and performed better in terms of explained variance for both monthly and seasonal precipitation predictors. However, for nRMSE, the models spanning the whole of Germany performed best (mean nRMSE=6.68%) (Table 2,Fig. 2a) compared to the lowland and mountain regions for seasonal precipitation. Models applied in the alpine region displayed the highest variation in R^2^ values (Table 2, Fig. 2b). The models from all regions varied significantly from each other in terms of RMSE (Wilcoxon Test: Table 4, Fig. 2a,b), except for tests of difference that showed a lower percentage of significance (Wilcoxon test: 68.75%, *p* < 0.05) between mountain and Germany regions with seasonal precipitation. The models applied to Germany in its entirety performed well. The contribution of both mountain (area=164,544 km^2^ (46%)) and lowland region (area=153,952 km^2^ (43%)) dominates the landscape (89%) of the entire area of Germany (355,926 km^2^). Therefore, the lower performing models applied in the alpine region (area=37,430 km^2^ (11%)), do not seem to have an influencing factor on the overall performance of the models applied throughout Germany, presumably due to their low contribution to the average. Accordingly, the seasonal precipitation model applied throughout Germany performed best and was therefore used to predict daily stream flow as input data for the IHA metrics.

### Modeling method

The first step in the modeling process was to extract the daily stream flow data from the gauging sites on a day by day basis for the entire study area (i.e. first day 1^st^ Jan 1950). All gauging sites with discharge data available for the same day (n=varies dependent on day) were used as the response variable input for the model of that specific day. A linear model was performed to estimate the stream flow for that particular day only. This procedure was repeated 23,376 times from the first day (1^st^ Jan 1950) until the last day (31^st^ Dec 2013) to create the daily time series of 64 years. In other words, the discharge is predicted on a daily basis for all of the study area. Unlike common hydrological modeling approaches, discharge predictions here are performed on the spatial dimension (i.e. as raster layers). Discharge time series are later aggregated by stacking the daily spatial predictions (i.e. stacking gridded datasets). We acknowledge that, although daily stream flow is used as the response variable in the model, we use a coarser (seasonal/monthly) resolution to predict the high resolution (daily) time series. We understand that achieving high accuracy from this type of model input is challenging. However, these data are readily available for the spatial scale and resolution we input into the model and were tested to provide adequate precision for use in ecological predictive models.

Exploratory analysis of the data showed that the distribution of the empirical stream flow data was heavily tailed due to several outliers, which violated the assumption of normal distribution. Therefore, a robust linear model (lmRob), less sensitive to a non-normal distribution^30^, was applied and implemented in the Robustbase R package^31^. This method uses maximum likelihood estimation to apply a weight system reducing the impact of outliers, while still including the benefits of simple linear regression and has been effectively used previously (Venables, W.N, personal communication, 2017). However, the performance metrics to determine goodness of model fit (e.g. R^2^) are easier and more intuitive to extract from simple linear models. Therefore, the weights were first calculated through the lmRob function and later introduced into a simple linear model to produce a linear model with weights (LMWW)^32^. The lmRob function calculates the weights using the method of MM-estimation, a development of Huber’s M-estimation^33^, which returns highly robust and efficient estimators. Further descriptions are outlined in Yohai (1987)^34^. For validation purposes, the spatial predictions from the LMWW were compared, using Wilcoxon tests on the RMSE, to those of the lmRob and no difference between models was found (Table 5). The resulting weighted linear regression (LMWW) is described in equation 1:

(1)Qs =∑s=1nWs(Qs−a−βxs)2

where *Q*_*s*_ is the discharge, *x* is the predictor, *W* is the weight at the *sth* gauging site, *α* is the intercept and *β* is the slope of the model. The configuration of gauges with stream flow data varied daily, therefore the calculated weights also differed daily.

### Application of the model

The model was used to extrapolate predicted stream flow values to each 1 km grid cell on the stream network raster of Germany (n = 85,363). This was done for every day in the time series from 01/01/1950 to 31/12/2013 (n = 23,376), creating a 64 year dataset of daily stream flow data, covering the entire stream network of Germany. Due to the nature of linear models, daily stream flow predictions included a number of negative discharge values, particularly in the headwater region of the stream network, where stream flow is lowest (for more details see “Limitations”). In the final dataset, any negative stream flow predictions were removed and replaced with the minimum predicted value across the entire time period for that grid cell. Next to the adjusted dataset, an additional dataset of the negative values is provided.

## Code availability

The R scripts are available online from www.github.com/ksirving/stream_flow.

There are three scripts in total, used for different steps in the modeling procedure.

1. The weighted linear regression model script is used to predict stream flow in any geographical region, given the appropriate data is available. The user needs to provide a data frame with daily stream flow input data for that specific region (e.g. GRDC www.bafg.de/GRDC/) and precipitation data as a GeoTIFF file that can be downloaded from www.earthenv.org/streams^17^. The output is a dataframe for each day of the simulation time containing stream flow values for all grid cells.
2. Format stream flow data calculated in script (1), including replacement of the negative values with the minimum value for that grid cell and structuring the data frame for input into IHA calculations and calculation of the IHA metrics.
3. Format and structure the provided stream flow NetCDF files for input into IHA calculations and calculation of the IHA metrics.

For script (2) & (3), the user needs to download the necessary IHA functions from www.github.com/USGS-R/EflowStats (2016)^10,11^ .

## Data Records

The modeled stream flow (m^3^ s^−1^) dataset contains daily (n = 23,376) data over 64 years (1950–2013) for every 1 km grid cell (n = 85,363) in the German stream network. The dataset is available as NetCDF files and is available for download (Data Citation 1). Each individual raster layer represents one day in the time series, which are available as annual raster stacks (n = 64). The user can subset the time series to the required period and follow script (3) to structure and format the data for input into the IHA calculations. All negative values have been replaced. However, annual NetCDF files (n = 64) containing all original values are provided as an additional dataset. Here, each individual raster layer (n = 1,621) of the NetCDF file represents the day in the time series and contains only the negative values. The 53 IHA metrics that were validated successfully are also available for download (Data Citation 1) for the same stream network (Germany, grid cells n=85,363). The IHA metrics are available as GeoTIFF files, with each layer representing one metric. All NetCDF and GeoTiff files are in WGS84 coordinate system with an extent of 55°N to 47°S latitude and 5°E to 15°W longitude. All layers contain 914 rows and 1,100 columns. To reduce the file size, all values have been multiplied by 10,000 in order to achieve an integer format without precision loss. Potential users therefore need to convert data back to the original units by dividing each raster file by 10,000 (data type = Int4S, NoData value = −999).

## Technical Validation

### Spatial Validation

To validate the newly-developed dataset (Data Citation 1), we split the flow data of each individual model (n=23,376 days) spatially into 70% training and 30% testing data sets. Each model was then built using the training data and then assessed on how well it predicts the independent testing data.

Wilcoxon tests were then applied to test for any difference between the root mean square error (RMSE) values of the training and testing datasets. The RMSE is a measurement of the model’s predictive ability. RMSE is an absolute measure of fit, calculated through the comparison of the observed and predicted stream flow values from corresponding sites (equation (2)):

(2)RMSE=∑i=1n(yobs,i−ysim,i)2n

where n is the number of sites, *y*_*obs,i*_ is the observed discharge value at the *ith* site and *y*_*sim,i*_ is the simulated discharge at the *ith* site.

RMSE is reported in the same units as the response variable (i.e. m^3^ s^−1^) and is an important measurement of fit when the model is used for prediction. Zero indicates the best possible fit. The RMSE was calculated through the HydroGof package^29^.

An indication of good model performance was considered to be the lack of significant differences between the RMSE values of the training and testing datasets. A method of subsetting the large dataset into yearly subsamples was applied (n = 365 ^∗^64). Each set of 64 Wilcoxon tests are reported as percentage (%) of tests that show a significant difference.

According to the RMSE comparison between the training and testing datasets, the majority (95%, n = 61, *p* > 0.05)) of tests showed no significant difference between datasets (Table 5, LMWW: % of significance). The three years that showed a significant difference are listed in Table 6.

### Calculation and validation of hydrological metrics

There are 171 IHA metrics that describe the frequency, magnitude, duration and timing of stream flow events set out in Olden & Poff, (2003)^19^ (full descriptions in Supplementary Table 1 in the supplementary material). Of these, 165 were chosen and a correlation analysis performed. The six that were omitted included drainage area variables, such as “mean annual runoff”, an aspect which is beyond the scope of this study. The hydrological metrics were calculated using the functions available from the R package EflowStats (www.github.com/USGS-R/EflowStats)^10,11^. For validation, the observed data were randomly and spatially split into 70% training and 30% testing datasets. The model was built using the 70% training data, with its subsequent predictions calculated for the 30% testing data, which were used as input for the metrics calculation of the simulated data. Any simulated values that were below zero were replaced with the lowest value throughout the time series for that grid cell.

A continuous (gapless) daily time series was needed to calculate the IHA metrics. Due to the nature of the data splitting, the testing dataset was not continuous over the entire time series for every gauge. To test the most truthful values of observed stream flow, instead of interpolation, and to reduce computing time, this required subsetting the observed flow data into a smaller dataset including 518 gauging sites, across 20 years (1965–1984). This subset was then compared with metrics calculated from the above described simulated flow data for the same time period and grid cells matching the gauge sites in the observed dataset. Julian day and hydrological year information were added to each dataset, which were then arranged to match the format outlined through EflowStats. The 165 metrics calculated from the observed and simulated stream flow data were then compared using Spearman’s Rank correlation coefficient (r). The specific metrics that were correlated (r = >0.5) were calculated for all grid cells in the stream network (n=85,363).

**Figure 3:**
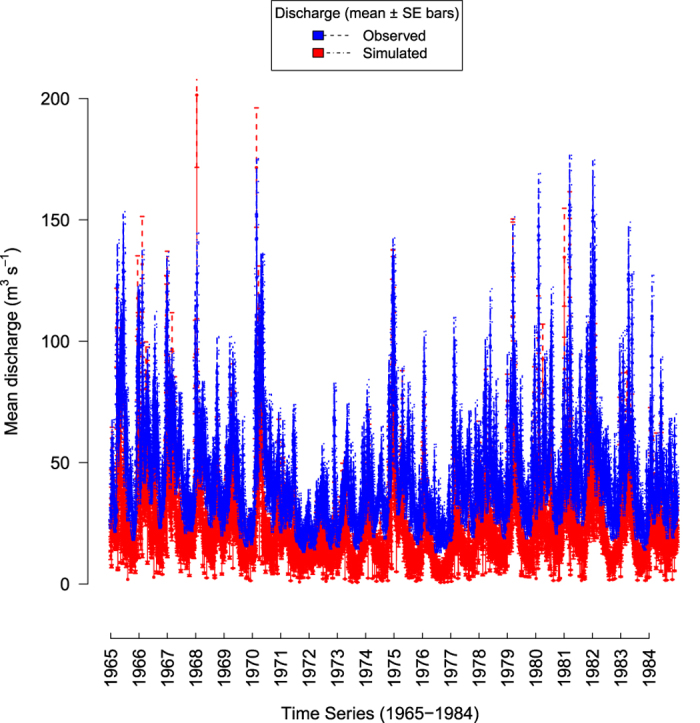
Time series of observed and simulated daily mean discharge. Taken over time period of 20 years (1965–1984), at gauge sites±SE bars (n = 518 gauges).

Spearman’s correlations of the observed and simulated hydrological metrics for 518 sites are set out in the supplementary material (Supplementary Table 1). Of the 165 metrics, 53 were sufficiently (r >= 0.5) positively correlated with their observed equivalent. Positive correlations were predominantly found for metrics describing mean values of stream flow, e.g. mean monthly flow. Figure 3 shows the mean stream flow for the same 518 sites and time period (1965–1984). While the simulated stream flow was consistently lower than the observed one throughout the time series, there is a good match in the recurring temporal trends of the stream flow. It is this match in the long term trend which indicates that the stream flow predicted here can be used to derive hydrological metrics for large spatial scales, at high spatiotemporal resolutions.

### Temporal validation

To validate the predictions temporally, the data were split as above and Kling-Gupta Efficiency (KGE)^35^, RMSE and the coefficient of determination (R^2^) were calculated on the observed vs. simulated values in the testing dataset, across the entire time series of 64 years through the R package HydroGof^29^.

From a total of 1,014 sites, 69 had KGE over 0.6 (mean = −1.58, n = 1,014) and 352 had R^2^ over 0.5 (mean = 0.4, n=1,014). In addition, RMSE for the testing data shows a mean of 29.67 (m^3^ s^−1^) and the training data a mean of 29.67 (m^3^ s^−1^). Full values are shown in the supplementary material (Supplementary Table 2).

We are aware that the majority of these results do not meet the requirements for most hydrological applications^36^. However, the objective of this study was to produce streamflow data to be used for ecological predictive models, therefore we performed GLM models on metrics calculated with both observed and simulated values.

**Figure 4:**
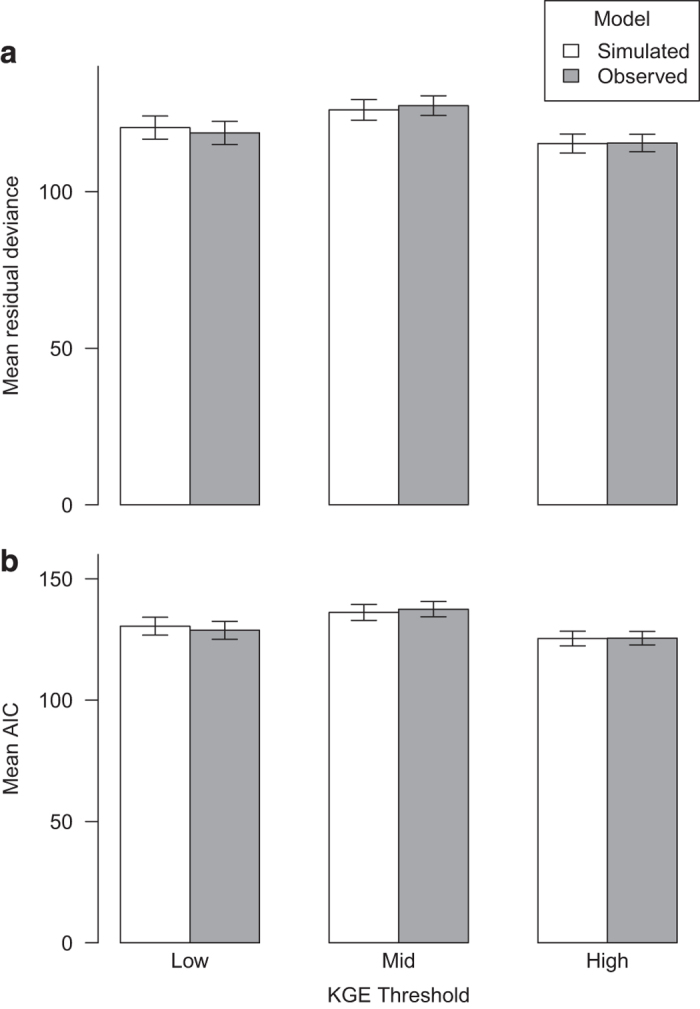
Comparison of observed and simulated GLMs across 3 KGE thresholds. Low (KGE < 0, no of sites= 108), mid (KGE >0<0.4 no of sites = 116) and high (KGE > 0.4, no of sites = 103). The columns represent (**a**) mean deviance, (**b**) mean AIC, on total n=34 species, sub-divided per KGE category; low (n = 20), mid (n = 32), high (n = 27) with standard error bars.

For this purpose, benthic macro-invertebrate occurrence data for Germany were collected from federal state environment agencies (Umwelt Bundesamt UBA) database (see supplementary material for full species list, Supplementary Table 3). Species presences were defined as those with at least one occurrence per site over the period 2005–2013 and occurring at more than 19 sampling sites across all Germany. The gauging sites were paired with species sampling sites in QGIS^22^ within a buffer of 3 km. To ensure the sample sites were placed on the original stream, the original flow accumulation value had to be within 10% of the flow accumulation of the newly allocated grid cell^37^. A total of 327 sites remained that had associated observed and simulated discharge, as well as species presence data. A total of 34 species with over 20 presences were modeled. The gauging sites were split into 3 categories of ranging KGE values: low (KGE < 0, no. of sites= 108, no of species = 20), mid (KGE > 0 < 0.4 no of sites= 116, no of species= 32) and high (KGE > 0.4, no of sites= 103, no of species = 27). Two sets of four IHA metrics (TA1, TA2, MH21, MH8, see Supplementary Table 1 for full descriptions^19,38,39^) were calculated with observed and simulated discharge, respectively. For each KGE category we performed a GLM to predict species distribution for Germany. The comparison of the skill, which is defined as the residual deviance and Akaike Information Criterion (AIC), from each GLM is illustrated in Fig. 4 with full results shown in the supplementary material (Supplementary Table 3), where lower values indicate a better fit. The validation of the ecological models showed that the simulated stream flow yielded almost the same model accuracy as the ones calculated with observed stream flow. A Wilcoxon test was applied as a statistical test for difference on both the AIC and the residual deviance. Overall, there was no difference between models applied with observed or with simulated predictors (low: Wilcoxon test, *p *= 0.69; mid: Wilcoxon test, *p *= 0.85; high: Wilcoxon test, *p *= 0.96). The results of the GLM suggest that the data predicted from this study is adequate for the purpose of ecological predictive modeling at the scale of Germany, encompassing steep environmental and hydrological gradients that facilitate the model to discriminate between suitable and non-suitable hydrological conditions for the species considered here.

## Usage Notes

Obtaining stream flow data for ecological modeling over large scales with high spatial and temporal resolution is challenging in terms of data availability and computing effort. Our simple regression model was able to overcome these challenges while still rendering adequate predictions regarding the occurrence of benthic macro-invertebrate species predicted using GLMs. The set of 53 IHA metrics provided as downloadable GeoTIFF files (Data Citation 1) describe important aspects of the flow regime and has the potential to be applied in a number of freshwater investigations such as predictive modeling (e.g. SDMs)^40^ that are relevant for conservation and river management plans. The 64 year time series of simulated daily stream flow data are also provided as downloadable NetCDF files (Data Citation 1). Thus, the user can calculate IHA metrics for any other time period within the 64 years provided for any catchment or any river section of the 1 km German stream network.

Available data from GRDC and EarthEnv^17^, together with the procedure provided through an R script, creates an accessible method for calculating both daily stream flow data and IHA metrics within any other geographical region where gauged streamflow data are available. This is especially helpful in areas with insufficient resources to implement complex models.

### Limitations

We note that the daily stream flow values are estimated assuming conditions where most of the discharge is driven by precipitation. Hence, the user should be aware of the hydrological processes that drive the streams within the study region of interest such as the influence of groundwater^41^ and soil infiltration processes. The user should also validate the results if applying the model in headwaters and during extreme events. From the preliminary analysis we note that our model worked very well when applied in lowland regions; however, the models applied within the alpine region performed least well.

Rivers in lowland regions are typically fed by ground water^41^. Through soil infiltration and groundwater processes, stream flow has a slower response to precipitation. As we applied a seasonal resolution of precipitation, the delayed response time is incorporated within the model. The relatively low number of gauges used within the alpine model (Table 1) compared with the mountain and lowland regions, may partially explain its relatively low performance. Another explanation could be that the highly varied topography creates a complex landscape and hence complex precipitation--run off interactions that are highly impacted by daily events. Here, attributes such as altitude and steep mountain slopes are major factors in determining stream flow regimes by distributing rainfall to streams much quicker than in lowland areas. In addition, a prominent feature of alpine regions is the existence of glaciers and increased snow cover. These features largely control flow regimes through the periodical storage and melting of rainfall, which is released on various time scales from days to years^42^. This time lag, together with highly varied precipitation patterns^43^ and daily fluctuations in the melting of snow^44^, is not reflected in the seasonal precipitation of our model and is therefore not captured.

**Figure 5:**
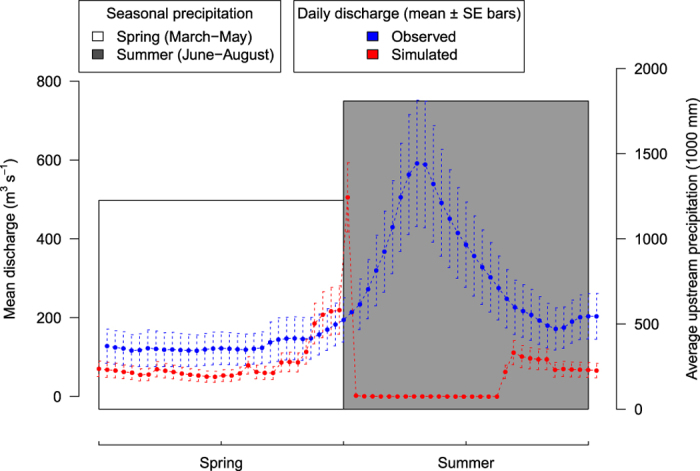
Time series of observed and simulated discharge (mean±SE bars) over lowland ecoregion in May & June 2013. Columns; seasonal precipitation value for that period, points; daily mean±SE bars observed and simulated discharge values.

The outliers that are apparent in Fig. 2b (lowland) represent low performing models (mean R^2^ = 0.003 +/− 0.0005, 1^st^–18^th^ June 2013). This time period coincides with an extreme flooding event in June 2013 ^45^ (Fig. 5). There was a very apparent peak in mean observed discharge (n = 77), which rose from 157.3 m^3^ s^−1^ on 27^th^ May to over 588.5 m^3^ s^−1^ on 9^th^ June (14 days). The outlier on May 31^st^ represents data from fewer gauges (n = 24) than available during the remaining days (n = 77 for all days), possibly because several stations temporarily ceased operating during the flood. It is evident that extreme flooding events are difficult to capture and predict in the lowland region with any modeling approach (Fig. 2). The flooding event in June 2013 was a result of heavy rainfall between 31^st^ May and 18^th^ June. This heavy rainfall was not reflected in the 50 year average precipitation value for that month in our model, which resulted in the model failing to converge, thus producing low performing predictions. In contrast, models applied in both mountain and Germany regions performed well for the same time period (mountain mean R^2^ = 0.99, Germany mean R^2^ = 0.99).

**Figure 6:**
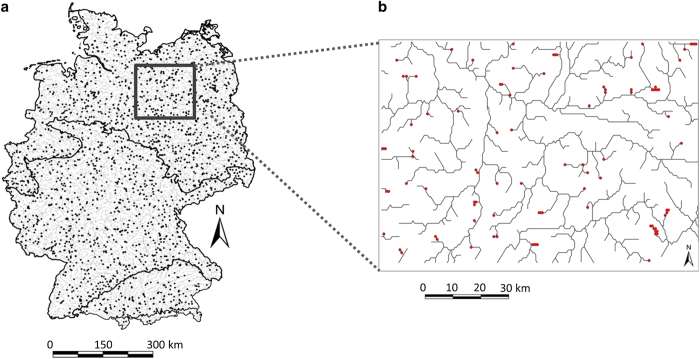
Distribution of predicted negative discharge values per grid cell. (**a**) all grid cells that show >5% negative values over the time series, (**b**) subsetted grid cells > 5% negative values over the time series. Total grid cells = 85 363; grid cells > 5% negative values over days in time series (n = 23,376) = 2,645 (red dots). Max % negatives = 6.93%.

A number of negative discharge values were predicted, which arise when the linear model produces a negative intercept, which corresponds to the value of flow when the precipitation is equal to zero. Predicted negative discharge values are reported as a percentage of all sites over the entire time series. The total number of negative stream flow values was 0.99% of the entire extrapolated dataset (n = 1 995,445,488 grid cells over the time series), considered a negligible amount. Overall, only 3.09% (n = 2,645) of all grid cells for the stream network of Germany (total n = 85,363) yielded more than 5% negative values (maximum for one cell 6.93%) over the entire time series (n = 23,376 days). The distribution of grid cells with negative flow values (Fig. 6a) showed a strong pattern towards the headwaters of the stream network (Fig. 6b). Although obvious, it is important to note that negative flow values are impossible and are to be understood as regions where no discharge is predicted. However, the simplicity of the model together with basic hydrological theory can, to a great extent, explain this issue. On occasions, the model produces a strong slope for the linear model, which could be induced by the difficulty of capturing the time lag of precipitation into rivers with high volume flow. The strong slope results from the direct association of flow with precipitation, without considering other hydrological processes such as (ground)water storage, evaporation and evapotranspiration from soil^46^, interception^47^ and surface depression storage^48^. If these factors were to be included, the model would likely show a non-linear response, e.g. causing zero flow for regions below a certain flow accumulation threshold. This could potentially decrease the number of negative flow values. However, such relationships are only possible with significantly more complex models and beyond the goal of our approach to use a simple model.

## Additional information

**How to cite this article:** Irving, K. *et al.*, A high-resolution streamflow and hydrological metrics dataset for ecological modeling using a regression model. *Sci. Data*. 5:180224 doi: 10.1038/sdata.2018.224 (2018).

**Publisher’s note:** Springer Nature remains neutral with regard to jurisdictional claims in published maps and institutional affiliations.

## Supplementary Material



Supplementary Table 1

Supplementary Table 2

Supplementary Table 3

## Figures and Tables

**Table 1:** Gauging stations.

Spatial region	Min	Max	Median
Alpine	6	112	97
Lowland	22	254	145
Mountain	43	584	418
Germany	71	944	685
Min, max and median number of gauging stations, caused by temporal gauge data availability, used in models throughout the time series (n=23,376 models) applied within each region.			

**Table 2:** Overall statistics.

	RMSE (m^3^ s^-1^)	nRMSE (%)	R^2^
*Flow Accumulation*			
Alpine	86.35±0.39	12.67±0.04	0.51±0.0027
Lowland	249.16±1.0	10.58±0.02	0.96±0.0005
Mountain	153.45±0.50	7.63±0.02	0.83±0.0016
Germany	173.69±0.59	7.44±0.01	0.87±0.0014
*Monthly Precipitation*			
Alpine	81.83±0.37	12.04±0.03	0.59±0.0027
Lowland	217.96±0.91	9.31±0.02	0.96±0.0005
Mountain	129.79±0.44	6.67±0.02	0.86±0.0014
Germany	154.62±0.53	6.68±0.01	0.90±0.0011
*Seasonal Precipitation*			
Alpine	81.63±0.37	12.02±0.03	0.59±0.0027
Lowland	218.48±0.91	9.34±0.02	0.96±0.0005
Mountain	138.82±0.47	6.95±0.02	0.86±0.0014
Germany	154.78±0.53	6.68±0.01	0.91±0.0011
RMSE, nRMSE & R^2^ of each model configuration (mean±SE, n = 23,376).			

**Table 3:** Wilcoxon test of difference of predicted discharge RMSE between predictors.

Spatial Region	FL x MP	FL x SP	MP x SP
Lowland	96.9*	96.9*	0
Alpine	14.1*	15.6*	0
Mountain	100*	100*	50*
Germany	98.4*	100*	0
Flow accumulation (FL), monthly precipitation (MP), seasonal precipitation (SP), and spatial region. Numbers indicate % of years (total=64 years, n=365 days) with significant difference (p < 0.05), overall significance when using total data set (n=23,376 days) indicated by *.			

**Table 4:** Wilcoxon test of difference of predicted discharge RMSE between spatial regions.

Predictor	AL x MM	AL x LL	AL x DE	MM x LL	MM x DE	LL x DE
Monthly precipitation	100*	100*	100*	98.4*	95.3*	95.3*
Seasonal precipitation	100*	100*	100*	96.9*	68.8*	95.3*
Alpine (AL), Mountain (MM), Lowland (LL), Germany (DE), and predictor. Numbers indicate % of years (total =64) with significant difference (p<0.05), overall significance when using total data set (n=23,376) indicated by *.						

**Table 5:** Spatial comparison (mean±SE) of regression model.

	RMSE (m^3^ s^−1^)	nRMSE (%)	R^2^	% of significance
*Training (70%)*				Training vs. testing
LMWW	154.21±0.55	6.89±0.02	0.83±0.0005	4.70%
lmRob	152.77±0.54	6.82±0.01	0.83±0.0005	
*Testing (30%)*				LMWW vs. lmRob
LMWW	151.61±0.58	7.35±0.02	0.82±0.0009	0%
lmRob	150.33±0.58	7.29±0.02	0.82±0.0009	
Training and testing data (70:30) and between model methods LMWW & lmRob; Wilcoxon test percentage of significance (%) of *p* < 0.05 concluding that the means are not significantly different (i.e. observed and predicted values are similar).				

**Table 6:** Wilcoxon test of significance.

Training mean	Testing mean	W value	P value	Method	Year
178.85	167.89	72922.00	0.03	lmRob	1950
222.04	214.89	72618.00	0.05	LMWW	1952
173.13	164.56	73816.50	0.02	LMWW	2000
194.89	182.15	74903.50	0.00	LMWW	2013
Training and test datasets of modeling methods; lmRob – robust linear model, LMWW – linear model with weights. Years that showed a significant difference.					
